# Dual targeting of EGFR signaling and microtubule dynamics enhances replication-associated cellular stress and suppresses tumor growth in gefitinib-resistant non-small cell lung cancer

**DOI:** 10.1371/journal.pone.0360164

**Published:** 2026-10-08

**Authors:** Mohamed El-Tanani, Shakta Mani Satyam, Sainath Prabhakar, Yahia El-Tanani, Ebrahim Safaii, Syed Arman Rabbani, Mark Sutherland

**Affiliations:** 1 RAK College of Pharmacy, RAK Medical and Health Sciences University, Ras Al Khaimah, United Arab Emirates; 2 Department of Pharmacology, RAK College of Medical Sciences, RAK Medical and Health Sciences University, Ras Al Khaimah, United Arab Emirates; 3 Department of Perfusion Technology, Manipal College of Health Professions, Manipal Academy of Higher Education, Manipal, Karnataka, India; 4 Royal Cornwall Hospital Trust, NHS, Truro, United Kingdom; 5 Department School of Chemistry and Biomedical Sciences, University of Bradford, Bradford, United Kingdom; University of Michigan, EGYPT

## Abstract

Acquired resistance to epidermal growth factor receptor (EGFR)-targeted therapy remains a major obstacle in the treatment of EGFR-mutant non-small cell lung cancer (NSCLC). This study investigated whether repurposing mebendazole (MBZ), a microtubule-disrupting agent, could restore sensitivity to gefitinib in gefitinib-resistant HCC827 GR5 cells. Gefitinib monotherapy showed minimal cytotoxicity, reducing cell viability only marginally (92.7 ± 1.5%), whereas MBZ significantly decreased viability to 79.7 ± 0.6% at 0.1 µM and 29.7 ± 0.6% at 0.5 µM (p < 0.001). Combination treatment (gefitinib 1 µM + MBZ 0.1 µM) further reduced viability to 37.7 ± 0.6% (p < 0.001). Mechanistically, alkaline comet assay revealed significantly increased single-strand DNA damage in the combination group (tail intensity 22.33 ± 0.56%, p < 0.001), while neutral comet assay confirmed elevated double-strand DNA breaks. MBZ also induced dose-dependent γ-H2AX activation (p < 0.001), supporting replication stress-associated DNA damage signaling. *In vivo*, combination therapy significantly suppressed tumor growth, prolonging tumor doubling time from 6.2 days in controls to 25 days and increasing growth delay to 18.8 days (p < 0.01), without substantial systemic toxicity. Collectively, these findings identify replication stress vulnerability as a therapeutically exploitable axis and support MBZ repurposing as an adjuvant strategy to overcome gefitinib resistance.

## 1. Introduction

Lung cancer has become a global public health problem with incidence and mortality figures continuing to rise year after year [1]. It is now the second most common cancer and the leading cause of cancer deaths worldwide, with 2.2 million new cases and 1.8 million deaths in 2020 accounting for approximately 15% of total cancer incidence and 25% of total cancer deaths [1,2]. There is a marked delay in diagnosis in most cases and even with the best of treatments, long term survival in patients with advanced lung cancer is very poor. The incidence and mortality of lung cancer show geographical and sex distribution with an increased and disproportionate burden of the disease in Asian populations and in males [3]. Approximately 85% of all lung cancers are non-small cell lung cancer (NSCLC) and this histological type of cancer is thus responsible for the bulk of the global lung cancer related mortality [4]. For all types of lung cancers, the 5-year survival rates have not improved in recent years. For the most common NSCLC the 5-year survival rate of about 25% has not improved over the last years. For small cell lung cancer, which is responsible for about 15% of all lung cancer cases, the 5-year survival rate of about 7% has not improved in recent years [5]. Although for NSCLC a variety of targeted therapy drugs as well as several checkpoint inhibitors have been introduced to the clinics, the 5-year survival rate of advanced stages of lung cancer has not changed and is approximately at 6% [6]. These numbers indicate that resistance to anticancer drugs is the major problem in lung cancer, not the onset of cancer.

Targeted treatments for lung cancer are designed to attack very specific characteristics of cancer cells. By targeting the cancer cells most specifically, these drugs have the potential to offer greater benefit and fewer side effects than many other cancer treatments. Recent years have seen significant progress in molecularly stratifying patients with NSCLC, thereby enabling identification of certain subgroups of patients who can be treated with targeted therapy. Of all patients with NSCLC, the greatest success has been achieved in the subgroup of patients with tumors that feature activation mutations of the epidermal growth factor receptor (EGFR) [7–11]. Approximately 10–15% of patients with Caucasian NSCLC and 40–50% of patients with Asian NSCLC harbor EGFR mutations in their tumors [10,12]. Most patients with EGFR-mutant NSCLC initially show marked and, in some cases, even dramatic responses to treatment with an epidermal growth factor receptor tyrosine kinase inhibitor (EGFR-TKI), such as gefitinib, erlotinib or osimertinib. In most cases, however, the clinical benefits of these treatments are short-lived and are eventually followed by clinical evidence of resistance to treatment. Importantly, resistance to EGFR inhibitors typically causes the tumor to switch from a state of EGFR dependency to a state of EGFR insensitivity. Thus, the greatest single challenge in treating lung cancer with targeted drugs is that of resistance to drugs. Resistance to EGFR-TKIs can involve several different changes within the cancer cell. First, secondary mutations within the EGFR gene, most notably T790M, have been identified in a subset of resistant cases [13,14]. However, most of the resistance is thought to be mediated by a system-level reprogramming of the cancer cell. To survive under continued targeted therapy, the cancer cell must reorganize its major signaling pathways, switch to alternative metabolic pathways, undergo an epithelial to mesenchymal transition, undergo epigenetic remodeling, and activate several bypass pathways including MET proto-oncogene receptor tyrosine kinase (MET) amplification, AXL receptor tyrosine kinase (AXL) up-regulation, and activation of the phosphoinositide 3-kinase/AKT serine/threonine kinase (PI3K/AKT) pathway [15–17].

Recently, replication stress homeostasis has emerged as a critical determinant of resistance to anticancer therapy. In tumor cells that are driven by an activated oncogene such as EGFR, chronic replication stress is a constant feature of cell proliferation. In response to EGFR inhibitors that do not kill EGFR-dependent tumor cells immediately, alternative means of coping with the impending catastrophe of aberrant cell cycle are generated by tumor cells to enable their survival under therapy. These stress-tolerating means to ensure persistent replication of tumor cells include stabilization of the replication fork, upregulation of deoxyribonucleic acid (DNA) repair functions, and protection of tumor cells from catastrophic genomic instability. Replication stress homeostasis is essential for the survival of cancer cells [18–20]. For cancer cells to sustain their proliferation even after the treatment with EGFR inhibitors, the cells must acquire the ability to allow the progression of the replication forks under the treatment. The ways of achieving this include activation of DNA damage response (DDR), upregulation and/or activation of the enzymes that are involved in DNA replication and repair, as well as the changes in cell cycle. Cancer cells that are unable to achieve these ends suffer from a catastrophic level of genomic instability leading to cell death. Therefore, targeting replication stress tolerance in cancer cells will kill the cancer cells.

Microtubules are integral to many cellular processes including mitosis, intracellular transport, cellular organization, and replication and transcription processes that require spatial organization. Inhibiting the polymerization of microtubules can cause cells to arrest in mitosis leading to chromosomal instability. Such stressed cells activate a variety of cellular stress response pathways [21–23]. Importantly, the processes of replication fork movement and of chromosome segregation during mitosis are also dependent on an intact microtubule structure as well as on several intracellular transport processes [24,25]. Mebendazole (MBZ) is a benzimidazole-class of anti-helminthic agents repurposed as anticancer agents due to their ability to bind to beta-tubulin (β-tubulin) thus interfering with microtubule dynamics. When compared to other chemotherapeutic agents targeting microtubules, MBZ possesses improved safety, oral bioavailability and anticancer activity. Although there are several reports on the mechanisms of action of MBZ, its effects on replication stress of EGFR-mutant and -inhibitor-resistant NSCLC cells have not yet been elucidated [26–28].

In our previous study, we demonstrated that combined targeting of EGFR signaling and microtubule dynamics using gefitinib and MBZ significantly reduced tumor cell viability and modulated stress-associated signaling pathways, including Interleukin-1 beta (IL-1β), Interleukin-6 (IL-6), Tumor necrosis factor-alpha (TNF-α), and Interferon-gamma (IFN-γ), as well as genes involved in nuclear transport and extracellular matrix remodeling [26]. These findings suggested that dual targeting disrupts tumor–microenvironment communication and intracellular stress signaling networks governing inflammation, mitosis, and cellular trafficking. However, that study was limited by using EGFR-independent models (A549 and MDA-MB-231), which do not reflect oncogene addiction or clinically relevant EGFR-driven resistance biology. A549 cells are primarily Kirsten rat sarcoma viral proto-oncogene (KRAS)-driven, while MDA-MB-231 cells represent heterogeneous triple-negative breast cancer lacking EGFR dependency. Consequently, those findings reflected broad cytotoxic and stress-modulatory effects rather than true reversal of EGFR inhibitor resistance.

To address this critical limitation, the present study focuses exclusively on HCC827 GR5 cells, a well-characterized gefitinib-resistant derivative of EGFR-mutant NSCLC. This model retains EGFR oncogene dependency while exhibiting clinically relevant acquired resistance, thereby enabling precise interrogation of adaptive survival mechanisms under sustained EGFR inhibition. This transition represents a conceptual shift from generalized cytotoxic pharmacology to precision resistance biology within oncogene-addicted systems. A further limitation in current therapeutic evaluation is reliance on static endpoint assays that fail to capture dynamic tumor adaptation under stress. Tumor response is an evolving process governed by replication stress accumulation, DDR activation, and time-dependent proliferative reprogramming. Therefore, integration of molecular DNA damage markers (phosphorylated histone H2AX at Ser139 (γ-H2AX)), comet assay-based single- and double-strand break (DSB) profiling, and *in vivo* tumor kinetic parameters such as tumor doubling time and growth delay is essential to define therapeutic efficacy beyond simple viability reduction.

Based on these considerations, we hypothesized that simultaneous inhibition of EGFR signaling and microtubule dynamics would generate a compounded cellular stress environment that exceeds the adaptive capacity of gefitinib-resistant NSCLC cells. Specifically, gefitinib-mediated suppression of survival signaling combined with MBZ-induced disruption of microtubule integrity was expected to amplify replication stress, destabilize replication fork progression, and induce a replication stress catastrophe state characterized by irreversible proliferative collapse and sustained tumor growth suppression. Accordingly, the primary aim of this study is to establish a mechanistic and systems-level framework linking EGFR signaling inhibition, microtubule disruption, and replication stress dynamics in gefitinib-resistant NSCLC. The specific objective was to determine whether dual targeting of EGFR signaling, and microtubule dynamics enhances replication-associated cellular stress and translates into sustained suppression of tumor growth *in vitro* and *in vivo*, while evaluating whether combination treatment produces a greater antitumor effect than either agent alone.

## 2. Materials and methods

### 2.1 Cell line and culture conditions

The gefitinib-resistant HCC827 GR5 cell line, a stable derivative of the human EGFR-mutant non-small cell lung cancer HCC827 cell line, was generously provided as a gift by Professor Pasi A. Jänne (Department of Medical Oncology, Dana-Farber Cancer Institute, Boston, Massachusetts, USA). The HCC827 GR5 cells retain the activating EGFR mutation present in the parental HCC827 cells but exhibit acquired gefitinib resistance through EGFR signaling rewiring and an enhanced ability to tolerate replication stress. To maintain the resistant phenotype, the GR5 cells were intermittently exposed to 1 micromolar (µM) gefitinib for approximately two weeks prior to the experiments to stabilize the resistant phenotype by reinstating drug-selective pressure and allowing the cells to return to a steady state of resistance-associated signaling. HCC827 and HCC827 GR5 cells were cultured in Roswell Park Memorial Institute-1640 medium (RPMI-1640) supplemented with 10% fetal bovine serum (FBS) and 1% penicillin–streptomycin at 37°C with 5% carbon dioxide (CO_2_). The cells were trypsinized using trypsin–ethylenediaminetetraacetic acid (EDTA) solution (0.25%) when they reached 70–80% confluence. The experiments were performed with early to mid-passage number cells (passages 5–20) to minimize phenotypic drift.

### 2.2 Drug preparation and experimental treatment conditions

Gefitinib is a specific inhibitor of the EGFR tyrosine kinase. MBZ is a drug that destabilizes microtubules. To target the replication of cancer cells from two different axes, we investigated the possibility of using gefitinib in combination with MBZ for the treatment of NSCLC. Gefitinib and MBZ were stored at −20°C in 100% dimethyl sulfoxide (DMSO) as stock solutions. On the day of the experiment, working solutions of the required concentrations were made up from the stock solutions in RPMI-1640 medium. The concentration of DMSO in medium was kept constant for all treatments at or below 0.1% to avoid solvent-induced cytotoxic effects. Following on from the setup of the cell cultures, the cells were then exposed to the respective drugs for set time periods. The end points of cell replication and/or cell viability were then determined for each treatment. A total of 5 different sets of treatment conditions were assessed. This included control (medium + 0.1% DMSO) and the single agent treatments of gefitinib (1 µM), MBZ (0.1 µM) and (0.5 µM). In addition, a range of combination treatments were assessed using gefitinib (1 µM) in combination with MBZ (0.1 µM) [26].

### 2.3 *In vitro* viability and cytotoxicity assessment

For the assessment of cytotoxicity, the cells were incubated with gefitinib, MBZ or a combination of both drugs for 72 h at 37°C in 96 well plates. The medium was then replaced by 5 mg/mL 3-(4,5-Dimethylthiazol-2-yl)-2,5-diphenyltetrazolium bromide (MTT reagent), and the cells were incubated for 3-4 h at 37°C. The formazan produced by viable cells was then dissolved by adding DMSO to the wells. The absorbance was read at 570 nm with 630 nm as the reference wavelength. For each data point the mean of the three wells was calculated and expressed as a percentage of the corresponding control value. All the experiments were done in duplicate and repeated three times. The plates were calibrated prior to use, and the intra-assay variation was determined by measuring the absorbance of the medium in the wells that were left blank. Cell viability was calculated using the equation [26,29–31]:


$$\begin{array}{c} \%\text{ of Viability }(\text{healthy cells})\;=\;(\text{Absorbance of treated cells}/\text{Absorbance of control cells})\;\times \text{ 100} \end{array}$$

For each data point, the average value of three replicate wells was determined from three independent biological experiments. As appropriate for all assays, also calibration and plate controls were measured and analyzed.

### 2.4 DNA damage assessment using comet assay

DNA strand break formation was assessed using the single-cell gel electrophoresis (Comet assay) to quantify both single-strand breaks (SSBs) and DSBs at the individual cell level [32–34]. HCC827 GR5 cells were seeded in culture dishes at a density of 0.6–0.8 × 10⁶ cells per well and allowed to adhere overnight under standard culture conditions. Following pharmacological treatments, cells were immediately processed for comet analysis to preserve endogenous DNA damage states and minimize artefactual lesion formation. Cells were harvested using gentle enzymatic dissociation with minimal exposure to trypsin–EDTA, followed by immediate washing in ice-cold phosphate-buffered saline (PBS). The resulting cell suspensions were maintained at low density and embedded in low-melting-point agarose (0.5–0.7%) on microscope slides pre-coated with normal-melting-point agarose. Slides were allowed to solidify at 4°C and subsequently immersed in freshly prepared high-salt lysis buffer containing detergent at 4°C overnight to remove cellular and nuclear membranes, yielding nucleoids composed of supercoiled DNA loops. For detection of SSBs and replication stress–associated lesions, slides were subjected to alkaline unwinding conditions (pH > 13). This alkaline environment facilitates DNA relaxation and enables detection of alkali-labile sites, replication fork stalling, and single-strand DNA breaks. In contrast, DSBs were evaluated under neutral electrophoresis conditions, which selectively preserve double-strand DNA architecture and allow detection of true chromosomal fragmentation associated with replication fork collapse and irreversible genomic instability. Electrophoresis was performed under strictly controlled voltage and time conditions specific to alkaline and neutral assays. Following electrophoresis, slides were neutralized, fixed, and stained with SYBR Green I. Fluorescent DNA migration patterns (“comet tails”) were visualized using fluorescence microscopy, and 50–100 randomly selected nuclei per experimental condition were analyzed using validated comet assay image analysis software. DNA damage was quantified using two complementary parameters: tail DNA (%) and tail moment. Tail DNA (%) was calculated as the proportion of fluorescence intensity in the tail relative to total nuclear fluorescence using the equation: (tail fluorescence/total fluorescence) × 100. Tail moment was defined as the product of tail length and tail DNA percentage, providing an integrated measure of both DNA migration distance and damage intensity. Alkaline comet assay signals primarily reflect early replication-associated stress, including SSBs and stalled replication forks, whereas neutral comet assay signals represent later-stage genomic instability characterized by DSB formation due to replication fork collapse. All analyses were performed in a blinded manner to avoid scoring bias. Quality control measures included strict monitoring of electrophoresis voltage, exclusion of overlapping or damaged comets, and maintenance of uniform staining and imaging conditions across all experimental groups.

### 2.5 γ-H2AX western blot analysis for DDR signaling

DDR activation downstream of replication stress was evaluated by quantifying γ-H2AX, a well-established marker of DNA double-strand break signaling and replication fork collapse [35,36]. For experimental consistency with comet assay conditions, HCC827 GR5 cells were seeded at a density of 0.6–0.8 × 10⁶ cells per well in 6-well plates and allowed to adhere overnight under standard culture conditions. Cells were subsequently treated for 24 h with gefitinib, MBZ, or their combinations as described in the experimental design. Following treatment, cells were washed with ice-cold PBS and lysed using radioimmunoprecipitation assay buffer (RIPA) supplemented with protease and phosphatase inhibitor cocktails to preserve protein integrity and phosphorylation status. Cell lysates were centrifuged to remove debris, and total protein concentration was quantified using standard colorimetric protein estimation methods to ensure equal loading across samples. Equal amounts of protein (normalized across all experimental conditions) were resolved by sodium dodecyl sulfate polyacrylamide gel electrophoresis (SDS-PAGE) and electro transferred onto polyvinylidene difluoride (PVDF) membranes under standardized transfer conditions. Membranes were blocked with 5% bovine serum albumin (BSA) in Tris-buffered saline (TBS) to prevent non-specific antibody binding. Membranes were incubated overnight at 4°C with rabbit monoclonal anti-γ-H2AX (Ser139) primary antibody (17 kDa; 1:3000 dilution). After washing, membranes were incubated with appropriate horseradish peroxidase (HRP)-conjugated secondary antibodies at room temperature for 1 h. Immunoreactive protein bands were visualized using enhanced chemiluminescence (ECL) detection. Band intensities were quantified using densitometric analysis software, and γ-H2AX expression levels were normalized to beta-actin (β-actin) as the internal loading control. All experiments were performed in biological triplicates, and exposure times were kept constant across all blots to ensure comparability between experimental groups.

### 2.6 *In vivo* xenograft model

A total of 80 BALB/c mice (6-8 weeks old, female) weighing 18-22 g were used for the establishment of xenografts as well as for treatment experiments. Animals were kept under controlled environmental conditions, i.e., 12 hr light / 12 hr dark cycles, temperature 22-24°C and humidity 40-60%. Animals also always had access to sterile food (standard rodent chow) and drinking water. All animals were acclimatized to the laboratory conditions for a period of one week prior to tumor implantation to minimize any pre-existing variations in physiological and/or metabolic parameters [37–40]. All experimental procedures were conducted in strict accordance with the institutional guidelines of Ras Al Khaimah Medical and Health Sciences University (RAKMHSU) and internationally accepted standards for the care and use of laboratory animals. The study protocol was reviewed and approved by the Animal Ethics Committee of RAKMHSU (Approval No. RAKMHSU-AEC-114-2024/25-F-M). All *in vivo* experiments were performed in compliance with the Animal Research: Reporting of *In Vivo* Experiments (ARRIVE) 2.0 guidelines to ensure transparency, reproducibility, and robust reporting of animal research.

The *in vivo* experimental design was divided into two studies, a short-term 11 days study (to study the pharmacological effects of the compounds in terms of DNA damage) and a long-term 40 days study (to study the effects of the different compounds on the growth of the tumors, studying the different stages that the tumors go through during evolution). HCC827 GR5 cells (1 × 10⁶ cells) were suspended in a 1:1 mixture of serum-free medium and Matrigel and injected subcutaneously into the flank region of mice. Tumor growth was monitored every other day using digital calipers, and two perpendicular dimensions (length and width) were recorded for each tumor. Tumor volume (V) was calculated using the standard ellipsoid approximation formula:

$\text{V }=\;(\text{Length }\times \;{\text{Width}}^{\text{2}})/\text{2}$. All animals were treated when the tumors reached a volume of 80–120 mm³ and then randomized within the specified volume limits to ensure that all groups started with tumors of similar size. Mice were randomized (n = 8 per group) into experimental groups including vehicle control, gefitinib monotherapy, MBZ monotherapy, and combination therapy.

For the short-term *in vivo* study, tumor-bearing animals were randomly allocated into six experimental groups (n = 8 per group) and treated for 11 consecutive days as follows: Group 1 served as the vehicle-treated control; Group 2 received MBZ (10 mg/kg/day); Group 3 received MBZ (30 mg/kg/day); Group 4 received gefitinib (50 mg/kg/day); Group 5 received a combination of MBZ (10 mg/kg/day) and gefitinib (50 mg/kg/day); and Group 6 received a combination of MBZ (30 mg/kg/day) and gefitinib (50 mg/kg/day). The use of two concentrations of MBZ in this study was to establish a threshold dose for the effect of MBZ on the microtubules and to determine the biologically active MBZ dose for subsequent combination experiments with gefitinib.

For the long-term *in vivo* study, tumor-bearing animals were randomly allocated into four experimental groups (n = 8 per group) and treated for 40 consecutive days as follows: Group 1 served as the vehicle-treated control; Group 2 received MBZ (10 mg/kg/day); Group 3 received gefitinib (50 mg/kg/day); and Group 4 received a combination of MBZ (10 mg/kg/day) and gefitinib (50 mg/kg/day). The high-dose MBZ (30 mg/kg) regimen was not included in the long-term *in vivo* experiments, as the study was specifically designed to evaluate pharmacologically relevant and translationally meaningful dose combinations rather than dose-escalation toxicity endpoints. The primary objective was to evaluate whether combining gefitinib with low-dose MBZ produced a greater antitumor effect than either agent alone at therapeutically relevant exposure levels aligned with chronic treatment settings and clinically tolerable dosing strategies. Accordingly, only low-dose MBZ (10 mg/kg) in combination with gefitinib (50 mg/kg) was selected for the extended study to ensure a focused evaluation of long-term tumor growth kinetics, minimize variability arising from dose-dependent systemic stress effects, and maintain experimental conditions conducive to interpreting the sustained antitumor effect of the combination. This approach allowed a more precise assessment of sustained anti-tumor efficacy under conditions that reflect realistic therapeutic constraints.

### 2.7 Tumor growth kinetics

Tumor growth was monitored every other day using digital calipers, and two perpendicular dimensions (length and width) were recorded for each tumor. To enable normalization of inter-individual variability, tumor growth was expressed as relative tumor volume (RTV), calculated as the ratio of tumor volume at a given time point (Vt) to the initial tumor volume at the start of treatment (V0), i.e., RTV = Vt / V0. Here, Vt represents tumor volume at time point t, and V0 represents baseline tumor volume prior to treatment initiation. Relative tumor volume doubling time (RTV2) was calculated based on the exponential growth relationship of RTV over time using the formula:

RTV2 = t × log2/log(RTVt / RTV0), where t represents the time interval (in days) between two measurements, RTVt is the RTV at the later time point, and RTV0 is the RTV at the earlier reference time point (typically baseline or previous measurement). Growth delay was defined as the difference in time required for treated tumors versus control tumors to reach a predefined tumor volume threshold, thereby providing a kinetic measure of treatment-induced suppression of tumor progression. Maximum body weight change was used as an indicator of systemic toxicity and was calculated as percentage change from baseline body weight using the formula:


$$\begin{array}{c} \text{ Body weight loss }(\%)\;=\;[(\text{Initial weight}-\text{Final weight})/\text{Initial weight}]\;\times \text{ 100}. \end{array}$$

At the end of the experimental period, animals were euthanized using an overdose of anesthetic agents in accordance with institutional ethical guidelines. Humane endpoints were predefined prior to study initiation and were based on tumor burden and clinical signs of distress. Animals exhibiting excessive tumor size or severe morbidity were humanely sacrificed before the scheduled endpoint to minimize suffering and ensure compliance with ethical standards.

### 2.8 Statistical analysis

Data were expressed as mean ± standard deviation (SD). Statistical analysis was performed using IBM Statistical Package for the Social Sciences (SPSS) Statistics software (version 32). Normal distribution of the data was checked prior to performing the parametric test. Statistical significance was determined using one-way analysis of variance (ANOVA) followed by Tukey’s post hoc multiple comparison test. A p-value < 0.05 was considered statistically significant. No formal pharmacological synergy model, such as the Chou–Talalay combination index, Bliss independence, Loewe additivity, or response-surface analysis, was applied because the experimental design did not include the concentration matrix required for such analyses. Accordingly, combination effects are interpreted descriptively and comparatively rather than as formally quantified pharmacological synergy.

## 3. Results

### 3.1 Integrated cellular response reveals graded collapse of proliferative capacity under dual-axis stress

Proliferating gefitinib-resistant HCC827 GR5 cells showed a dose-dependent decrease in cell viability after treatment with gefitinib or MBZ. The viability of untreated control cells was 100 ± 2.0% (mean ± SD), indicating that all cells were alive and that the assay was working properly. The untreated gefitinib-resistant cells showed a baseline level of cell viability of 92.7 ± 1.5%, indicating that these cells were resistant to gefitinib and that this drug was not able to induce a level of DNA damage in these cells that would kill the cells, even though this drug is able to inhibit the EGFR in these cells. Even at high doses gefitinib did not induce strong effects on cell viability in GR5 cells. At 1 μM the decrease in cell viability was marginal and only reached about 7% (92.7 ± 1.5%). In addition, no increased levels of γ-H2AX foci were observed in gefitinib-treated cells, indicating that no DNA damage was induced by gefitinib that could lead to a cell death response.

The effects of MBZ on the viability of gefitinib-resistant HCC827 GR5 cells were dose-dependent. A concentration of 0.1 µM caused a strong decrease in the cell viability to 79.7 ± 0.6% (p < 0.001), whereas a concentration of 0.5 µM caused a complete decline in the cell viability to 29.7 ± 0.6% (p < 0.001). This effect is typical for compounds targeting cell division and indicates a strong threshold effect. MBZ is assumed to destabilize microtubules. Partial disassembly of microtubules is sufficient to cause an increase in replication stress. In cells that are already stressed due to other reasons, this replication stress can be amplified to a degree that it becomes toxic for the cells.

Treatment of gefitinib (1µM) in combination with MBZ (0.1µM) resulted in a significant decrease in the viability of GR5 cells to 37.7±0.6% (p<0.001) that was comparable to that induced by the high dose of MBZ (0.5 µM) in the single agent study. These data show that partial destabilization of the microtubules by MBZ is sufficient to convert gefitinib-resistant cells into a replication stress-sensitive phenotype that can be killed by EGFR inhibitors (Fig 1).

**Fig 1:**
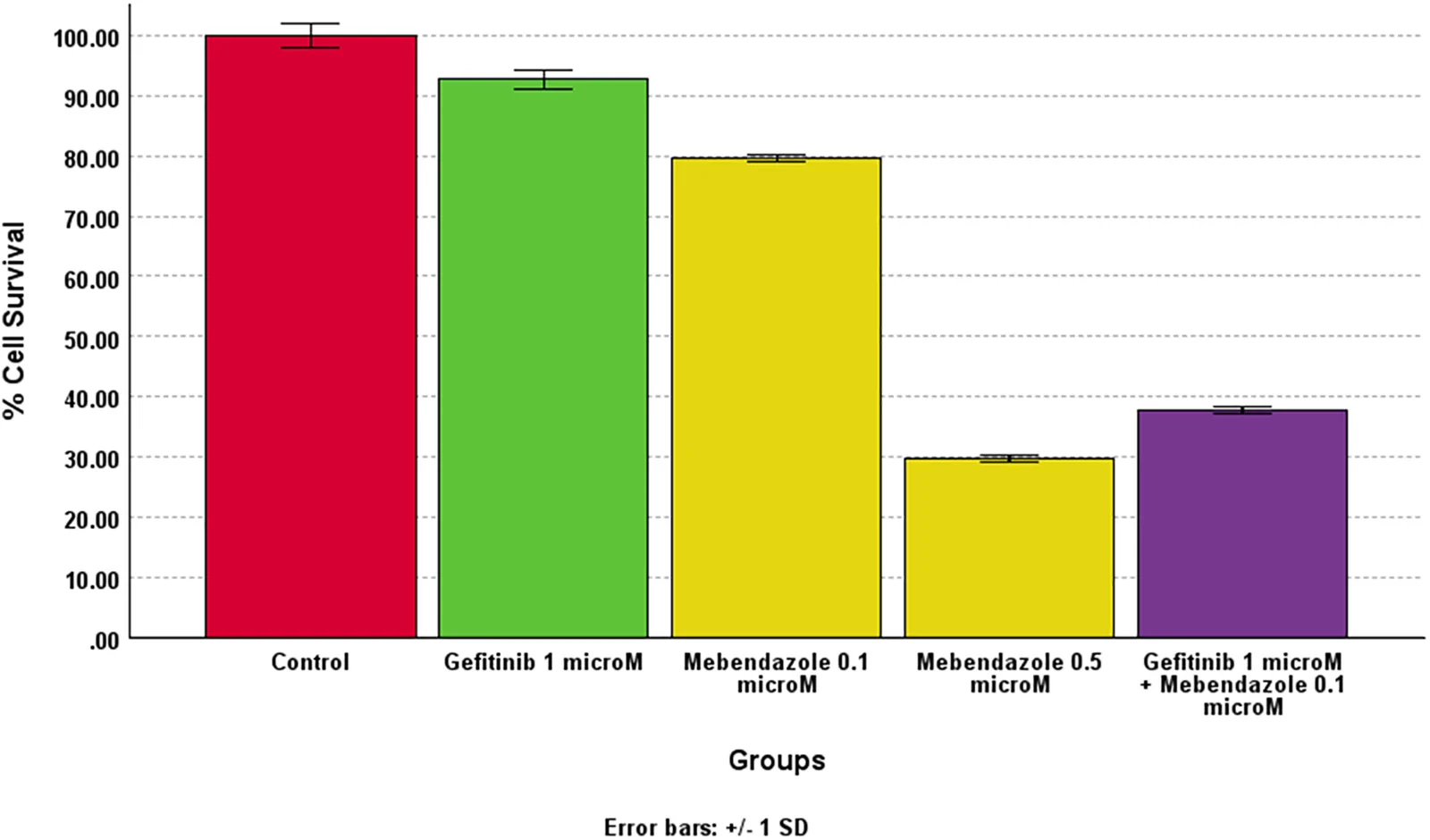
Dose-dependent effects of gefitinib and mebendazole on GR5 cell viability and combination-induced cytotoxic sensitization.

### 3.2 Single-strand DNA damage is amplified as an early replication stress signature

Alkaline comet assay analysis demonstrated that baseline DNA integrity in control cells remained stable, as evidenced by minimal SSB formation, with a tail intensity of 4.67 ± 0.54% and a tail moment of 2.33 ± 0.57, confirming low intrinsic genomic stress under standard culture conditions. Treatment with gefitinib resulted in a modest but statistically significant increase in SSB-associated parameters, with tail intensity rising to 12.67 ± 0.53% and tail moment to 4.33 ± 0.58 (p < 0.001), indicating limited replication perturbation despite effective EGFR inhibition.

MBZ treatment markedly amplified SSB formation in a dose-dependent manner at lower concentration, with 0.1 µM producing a substantial increase in tail intensity (20.33 ± 0.56%) and tail moment (9.67 ± 0.52), both highly significant (p < 0.001), consistent with pronounced replication fork instability. At the higher concentration of 0.5 µM, a relative reduction in SSB markers was observed (tail intensity 17.33 ± 0.54%), suggesting a mechanistic shift from accumulation of single-strand lesions toward increased double-strand DNA fragmentation due to replication fork collapse.

Combination treatment with gefitinib and MBZ produced the highest overall SSB burden, with tail intensity reaching 22.33 ± 0.56% and tail moment 9.67 ± 0.52 (p < 0.001), indicating an enhanced combination-associated increase in replication-associated DNA damage. Comparative statistical analysis revealed that tail intensity was significantly elevated across all treatment groups relative to control (p < 0.001 for all comparisons). Furthermore, tail intensity was significantly higher (p < 0.001) in the MBZ 0.1 µM group compared to gefitinib alone, while a reduction (p < 0.001) was observed at 0.5 µM relative to 0.1 µM. Importantly, this non-linear trend was observed only in alkaline comet parameters, while double-strand DNA damage did not show a corresponding decrease, supporting the interpretation that higher MBZ concentration is associated with a shift from detectable replication-associated single-strand lesions toward more extensive DNA fragmentation. Because replication-fork progression and restart were not directly measured, these findings should not be interpreted as direct evidence of replication-fork collapse. The combination group exhibited significantly higher tail intensity compared to both gefitinib and MBZ 0.5 µM groups (p < 0.001) and demonstrated a statistically significant increase compared to MBZ 0.1 µM (p = 0.012), supporting an enhanced combination-associated DNA-damage response under the conditions tested.

Similarly, analysis of SSB tail moment revealed a statistically significant increase across all treatment groups when compared with control cells (p < 0.001 for all comparisons), indicating a consistent induction of replication-associated DNA strand breaks following pharmacological intervention. Although gefitinib alone induced a measurable elevation in tail moment compared to control (p = 0.012), the magnitude of this effect remained comparatively modest, supporting its limited capacity to independently generate extensive replication fork instability in gefitinib-resistant HCC827 GR5 cells. MBZ monotherapy produced a marked escalation in tail moment at 0.1 µM (p < 0.001 versus gefitinib), reflecting pronounced replication stress and accumulation of single-strand DNA lesions associated with microtubule disruption–mediated mitotic perturbation. Importantly, a further increase in tail moment was observed at 0.5 µM MBZ when compared with 0.1 µM (p = 0.004), indicating a dose-dependent intensification of replication-associated DNA damage burden; however, this increase in magnitude must be interpreted in the context of damage-processing dynamics, where higher drug concentration may be associated with a shift from accumulation of single-strand DNA intermediates toward more extensive DNA fragmentation and altered lesion distribution rather than simple linear amplification of SSB accumulation.

The combination treatment group demonstrated the highest overall SSB tail moment values; however, statistical comparison revealed that the tail moment in the combination group was comparable to that observed in the MBZ 0.1 µM group, with no statistically significant difference between these two conditions. This finding suggests that the addition of gefitinib to low-dose MBZ does not further increase the absolute burden of detectable single-strand DNA breaks but instead stabilizes the system at a maximal replication stress threshold achieved by MBZ 0.1 µM alone. Mechanistically, this plateau effect is consistent with a saturation phenomenon in which replication forks already engaged in stress-induced stalling cannot generate proportionally higher SSB signals despite additional upstream signaling perturbation by EGFR inhibition. In this context, gefitinib likely contributes to replication stress priming rather than additive strand break formation, thereby enhancing cytotoxic efficiency without necessarily increasing measurable SSB endpoints beyond the MBZ-driven threshold (Figs 2 and 3).

**Fig 2:**
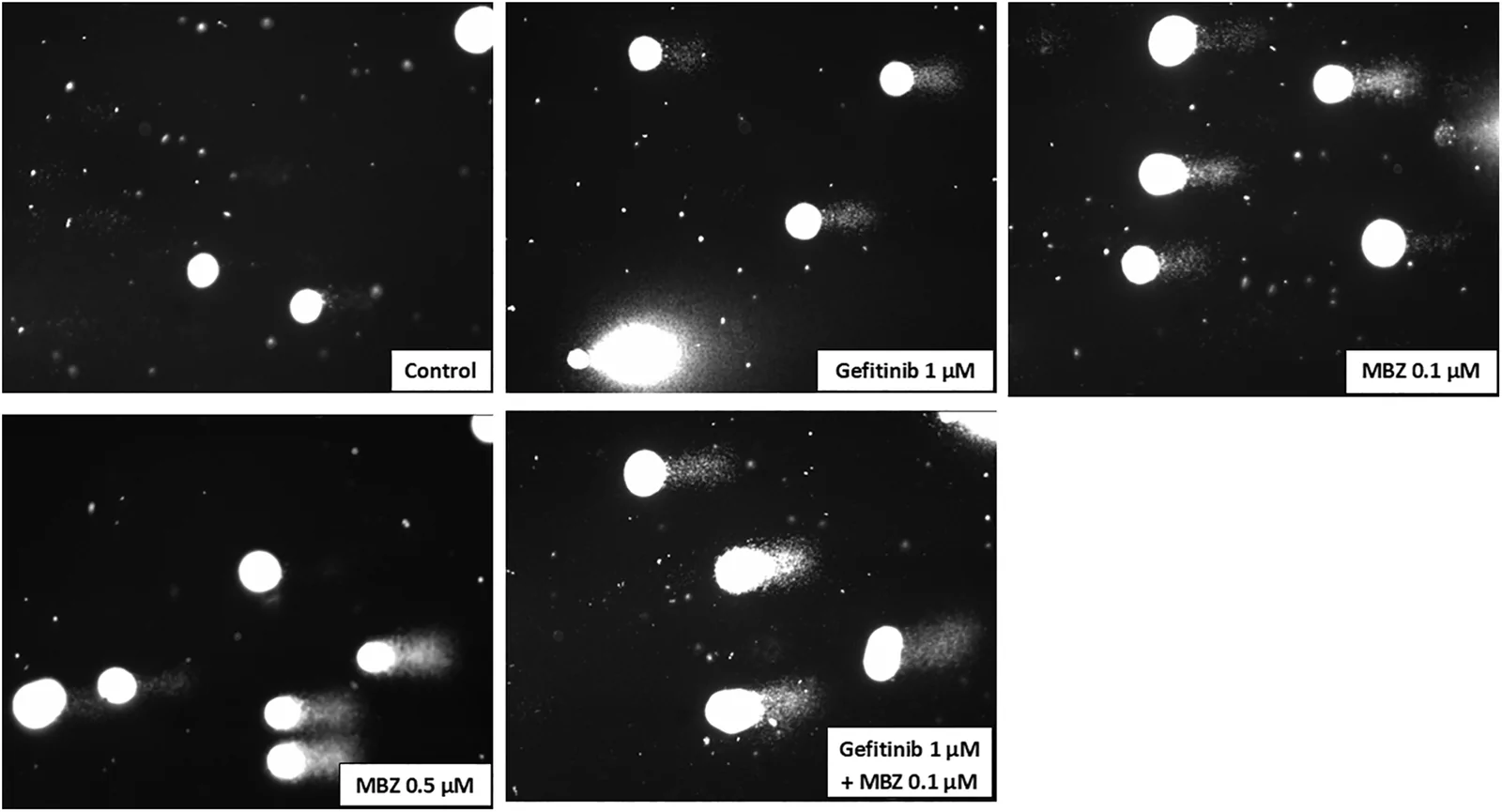
Representative comet assay images demonstrating single-strand DNA damage.

**Fig 3:**
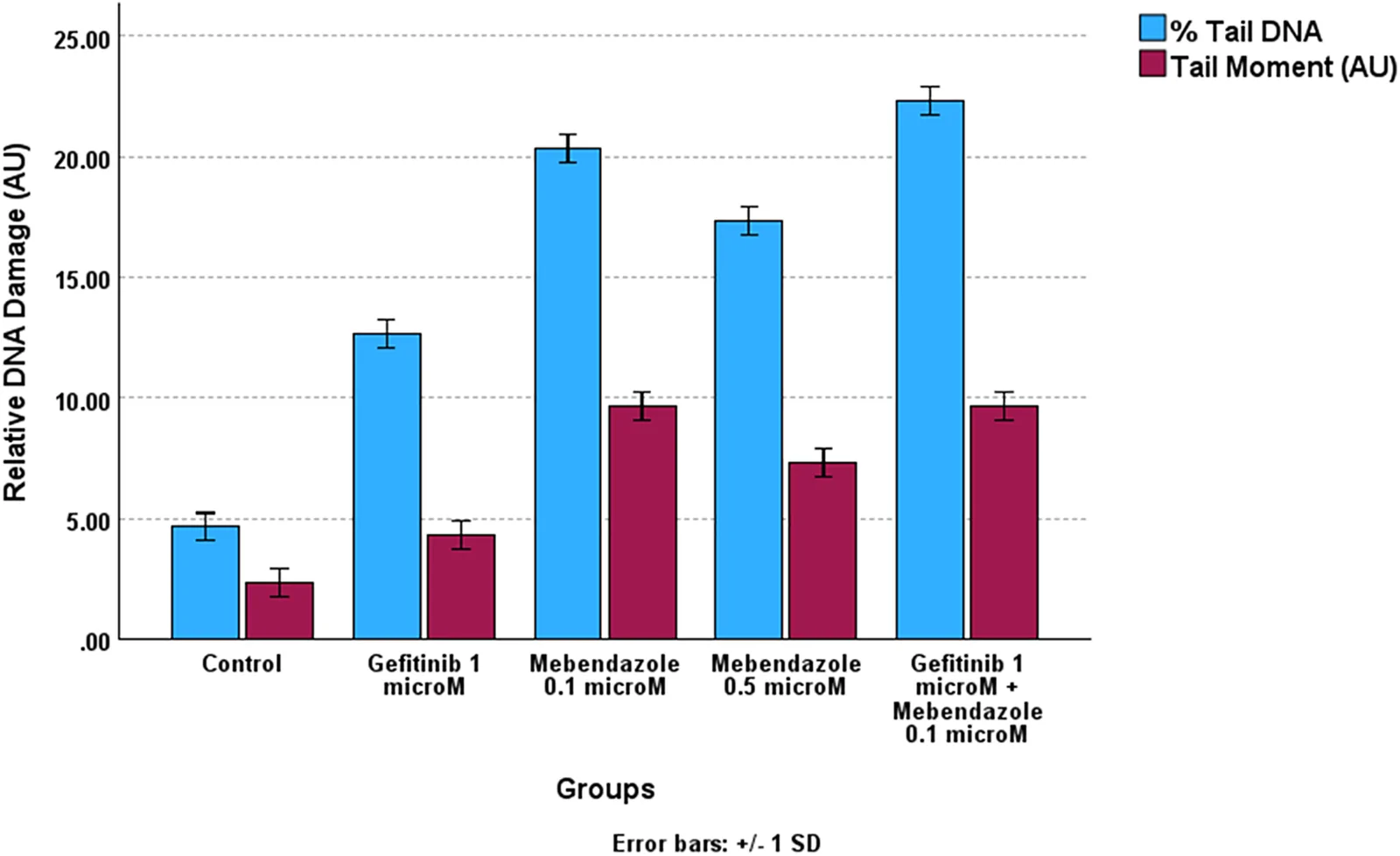
Quantitative analysis of single-strand DNA damage by comet assay.

### 3.3 Double-strand DNA damage reflects transition from replication stress to genomic collapse

Neutral comet assay analysis demonstrated that control cells exhibited low baseline levels of double-strand DNA breaks, as reflected by tail intensity (6.67 ± 0.57%) and tail moment (1.66 ± 0.54), confirming intact genomic stability and minimal endogenous replication-associated DNA fragmentation under standard culture conditions. Gefitinib monotherapy induced a statistically significant increase in DSB-associated parameters compared to control, with elevated tail intensity (11.65 ± 0.57%; p < 0.001) and tail moment (4.67 ± 0.55), indicating partial activation of downstream DNA damage signaling despite its limited cytotoxic efficacy in gefitinib-resistant HCC827 GR5 cells. This pattern suggests that EGFR inhibition alone generates sublethal replication perturbation that is insufficient to fully destabilize replication fork integrity but is adequate to produce measurable double-strand break signaling.

MBZ at low concentration (0.1 µM) did not produce a statistically significant increase in DSB tail intensity or tail moment when compared with control, indicating that at this dose the dominant lesion type remains confined to single-strand DNA breaks and replication fork stalling events without progression to overt double-strand DNA fragmentation. This finding is consistent with early-stage replication stress induction in which microtubule destabilization impairs mitotic progression, but cellular repair mechanisms still prevent conversion into irreversible genomic breaks. In contrast, higher concentration MBZ (0.5 µM) resulted in a pronounced and statistically significant increase in both tail intensity and tail moment (p < 0.001), consistent with a transition from replication-associated stress to more extensive DNA fragmentation. Because replication-fork progression and restart were not directly measured, these findings should be interpreted as being consistent with, rather than directly demonstrating, replication-fork collapse or failure of replication restart.

Combination therapy produced intermediate DSB levels (tail intensity 13.65 ± 0.58%; tail moment 5.65 ± 0.55), indicating a distinct mechanistic profile in which combined EGFR inhibition and low-dose microtubule disruption amplifies replication stress sufficiently to promote double-strand break formation without reaching the maximal fragmentation observed with high-dose MBZ alone. Statistically, DSB tail intensity in the combination group was significantly higher compared to gefitinib alone and MBZ 0.5 µM (p = 0.012 for both comparisons), whereas no significant difference was observed between the combination group and gefitinib monotherapy in terms of tail moment, suggesting that the overall spatial distribution and severity-weighted migration of DNA fragments were comparable in these two conditions despite differences in absolute break frequency.

Importantly, tail moment was significantly higher in the gefitinib group compared to MBZ 0.1 µM (p = 0.012), indicating that gefitinib induces a qualitatively distinct form of replication-associated DNA damage that contributes more to DNA migration dynamics than low-dose microtubule disruption alone at the level of double-strand break readout. Collectively, these findings suggest that DSB formation in this model is not a simple dose-dependent linear response but rather reflects a threshold-driven transition from replication fork stress (low-dose MBZ and gefitinib conditions) to fork collapse and genomic fragmentation (high-dose MBZ), while combination therapy operates in an intermediate mechanistic window characterized by replication stress amplification without maximal structural DNA disintegration (Figs 4 and 5).

**Fig 4:**
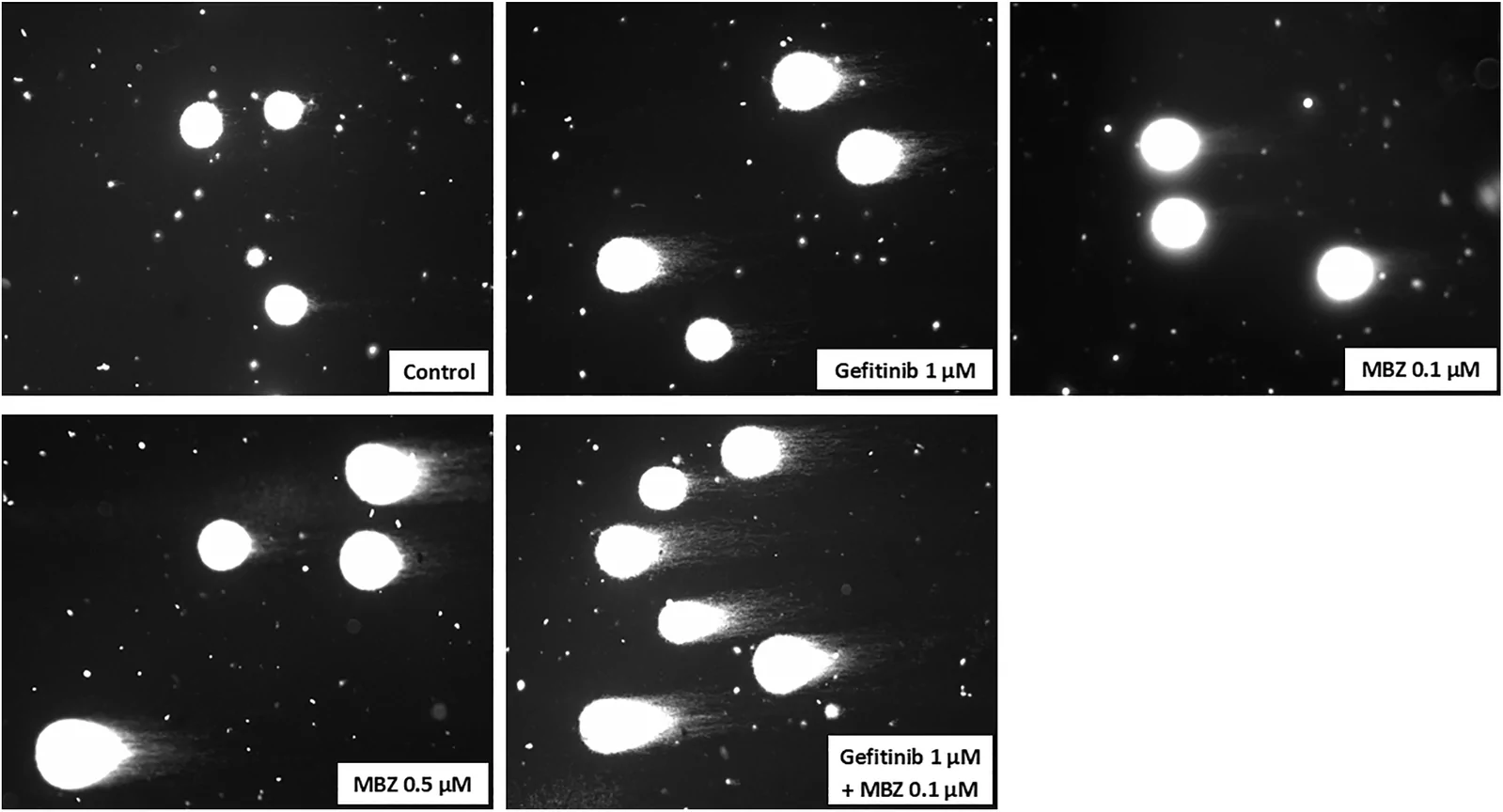
Representative comet assay images demonstrating double-strand DNA breaks.

**Fig 5:**
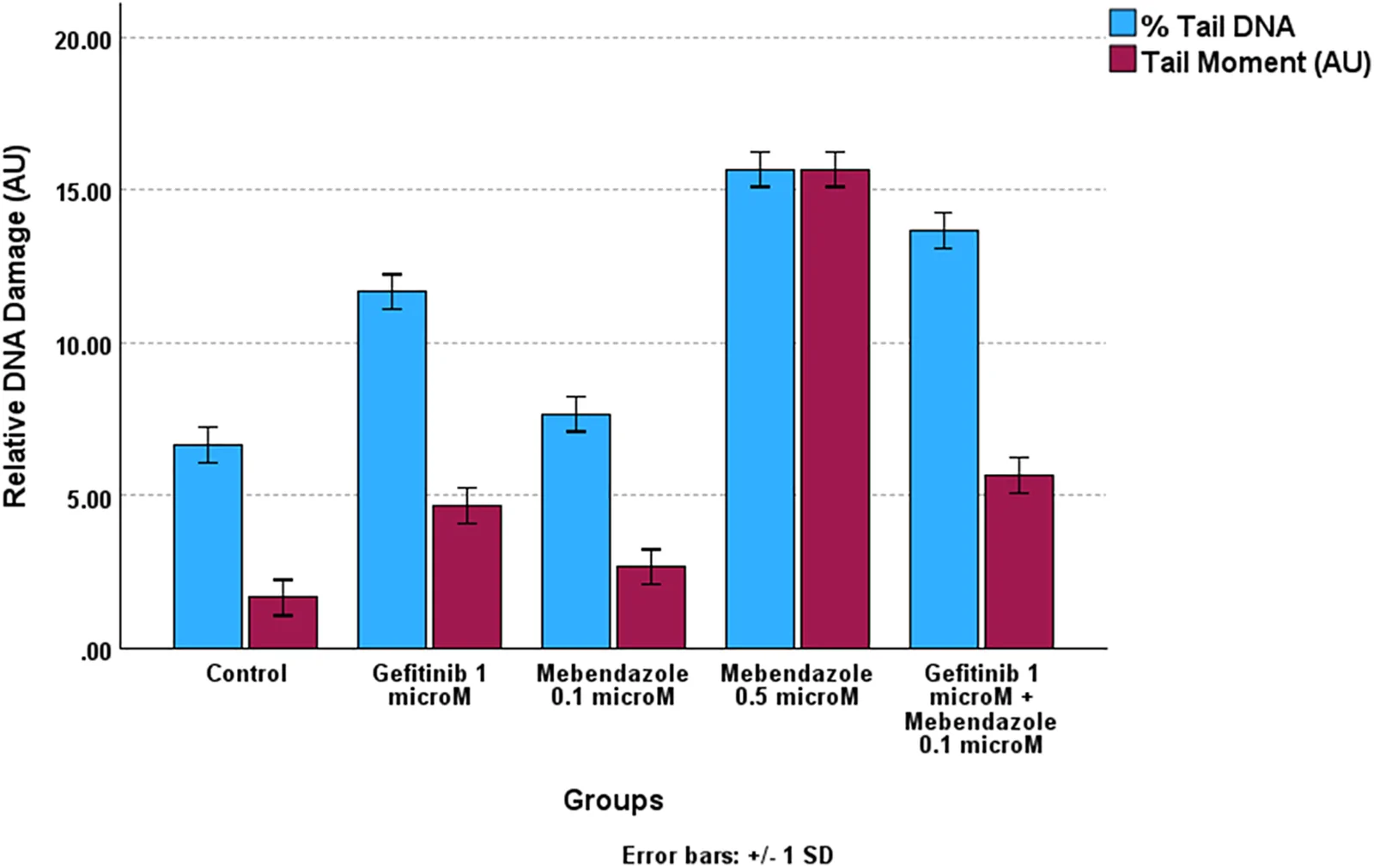
Quantitative analysis of double-strand DNA damage by comet assay.

### 3.4 γ-H2AX signaling reveals uncoupling between DNA break intensity and cytotoxicity

γ-H2AX expression analysis revealed no significant difference between control and gefitinib-treated cells, indicating that EGFR inhibition alone does not elicit substantial activation of the DDR pathway in gefitinib-resistant HCC827 GR5 cells under the experimental conditions. This suggests that gefitinib monotherapy induces only limited replication perturbation that remains below the threshold required for detectable γ-H2AX signaling and robust ataxia-telangiectasia mutated (ATM)/ataxia telangiectasia and Rad3-related (ATR)-mediated checkpoint activation.

In contrast, MBZ treatment produced a clear and statistically significant dose-dependent increase in γ-H2AX levels at both 0.1 µM and 0.5 µM (p < 0.001 versus control), demonstrating progressive activation of DDR signaling with increasing microtubule disruption. This pattern is consistent with increasing DNA-damage response signaling accompanying escalating cellular stress. Although replication-fork stalling or collapse may contribute to this response, these processes were not directly measured in the present study. The dose-dependent increase confirms that MBZ alone is sufficient to drive cells from a state of replication stress into overt DNA damage signaling.

Interestingly, combination therapy resulted in moderate γ-H2AX expression that was significantly lower (p < 0.001) than both MBZ monotherapy doses despite producing greater cytotoxicity. γ-H2AX is a marker of chromatin-associated DNA-damage response signaling and should not be interpreted as a direct quantitative measure of cell death. The dissociation between cytotoxicity and γ-H2AX intensity suggests that the enhanced loss of viability produced by the combination may involve cellular stress mechanisms that are not fully captured by the magnitude of γ-H2AX accumulation. The present findings are consistent with a model in which combined EGFR inhibition and microtubule disruption perturb replication-associated cellular processes and promote irreversible loss of proliferative capacity; however, direct replication-fork assays, checkpoint-activation measurements, and temporal analyses of DNA-damage resolution would be required to establish replication-fork collapse or checkpoint exhaustion mechanistically (Fig 6).

**Fig 6:**
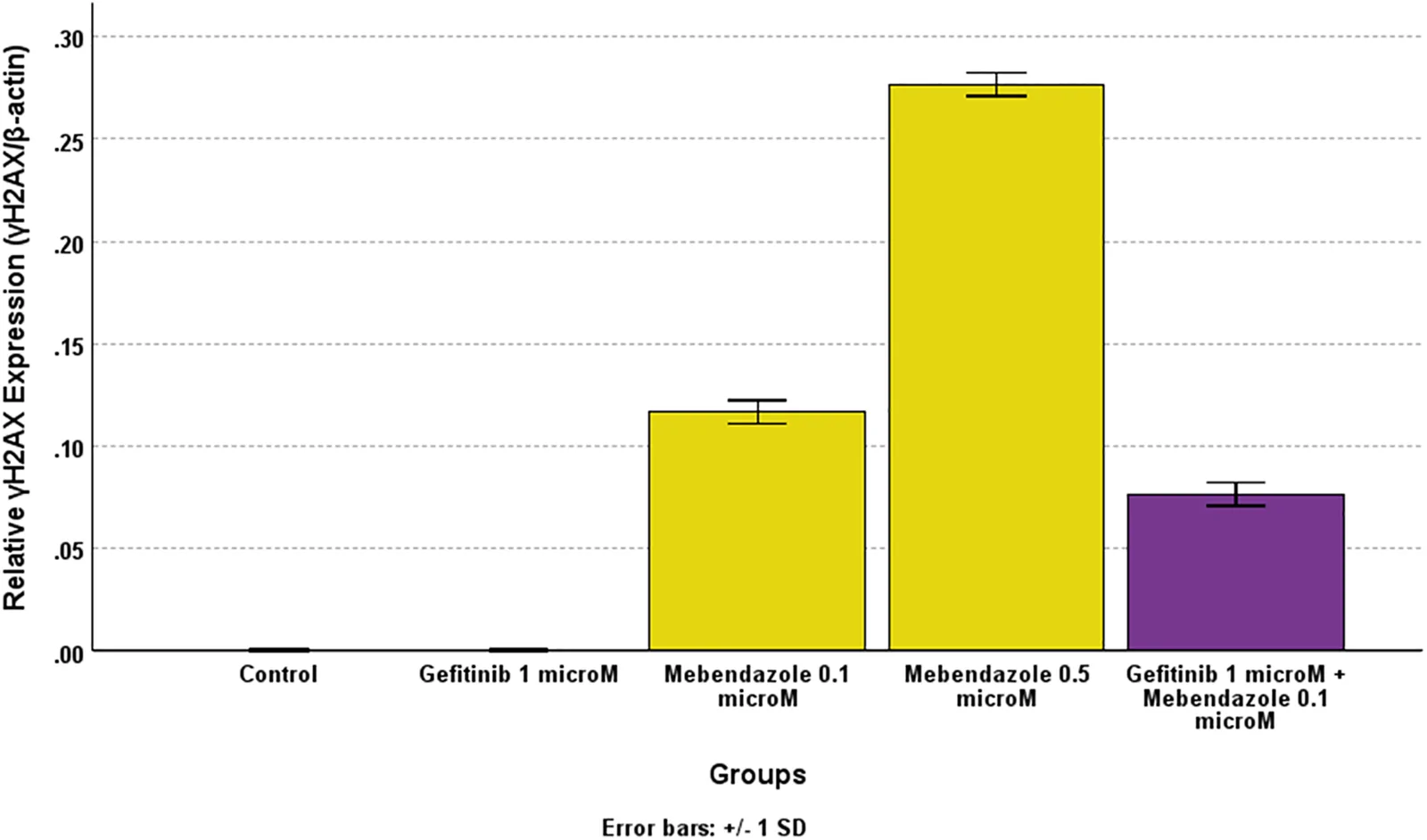
Effect of gefitinib and mebendazole on γ-H2AX expression in GR5 cells.

### 3.5 *In vivo* antitumor efficacy of mebendazole and gefitinib in HCC827 GR5 xenograft-bearing mice

#### 3.5.1 Short-term (11 days) tumor growth study.

Untreated control tumors exhibited rapid and continuous expansion throughout the study, with the RTV increasing progressively from baseline to approximately 3.63-fold by Day 11 (p < 0.001), confirming the highly proliferative nature of the HCC827 GR5 xenografts. MBZ monotherapy inhibited tumor growth in a dose-dependent manner (p < 0.05). Mice treated with 10 mg/kg MBZ demonstrated slower tumor progression than untreated controls, reaching a final RTV of approximately 2.54, whereas increasing the dose to 30 mg/kg further reduced tumor growth, resulting in a final RTV of approximately 1.69 (p < 0.01). These findings indicate improved tumor growth control with increasing MBZ dose during the early treatment phase. Gefitinib monotherapy (50 mg/kg) produced greater tumor growth inhibition than MBZ 10 mg/kg and achieved a final RTV of approximately 1.48 (p < 0.001), which was comparable to that observed with MBZ 30 mg/kg. Tumor growth remained substantially lower than that of untreated controls throughout the treatment period. Combination therapy produced the greatest suppression of tumor growth. The combination of MBZ 10 mg/kg and gefitinib maintained tumor volume close to baseline throughout the study, with a final RTV of approximately 0.96 (p < 0.001), representing the lowest tumor burden among all treatment groups. Similarly, the combination of MBZ 30 mg/kg and gefitinib markedly inhibited tumor progression, with a final RTV of approximately 1.10 (p < 0.001). The relatively small difference between the two combination regimens suggests that increasing the MBZ dose above 10 mg/kg provided minimal additional benefit when administered together with gefitinib (Fig 7).

**Fig 7:**
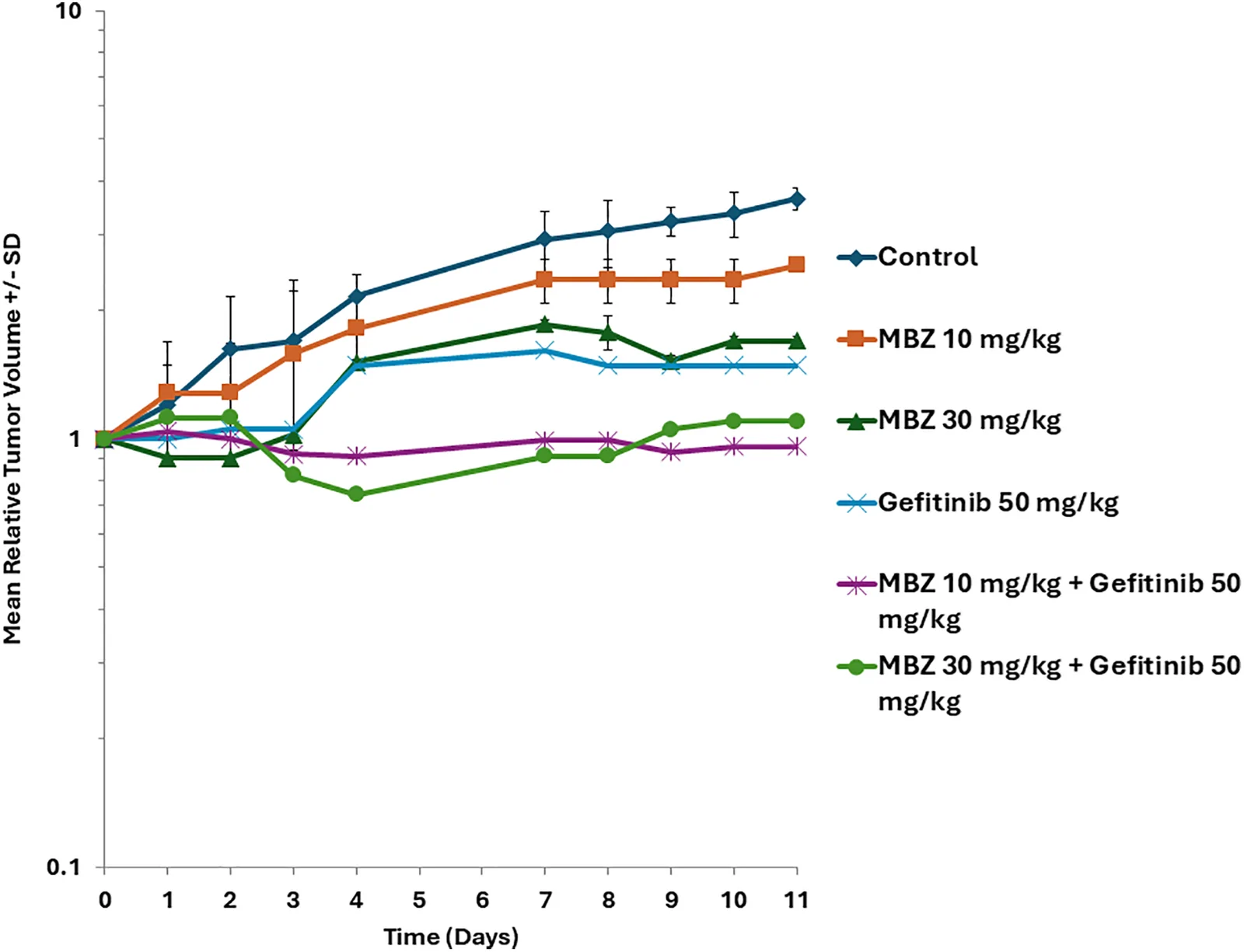
Short-term tumor growth inhibition following treatment in GR5 xenograft model.

Because the primary objective of this short-term study was to identify the minimum biologically effective MBZ dose capable of enhancing gefitinib activity, both 10 and 30 mg/kg doses were initially evaluated. The observation that low-dose MBZ combined with gefitinib produced tumor suppression comparable to the higher-dose combination supported the selection of the 10 mg/kg regimen for the subsequent long-term efficacy study. This experimental strategy was consistent with the *in vitro* findings demonstrating an enhanced cytotoxic effect of the combination at relatively low drug concentrations.

#### 3.5.2 Long-term (40 days) tumor growth study.

Untreated control tumors continued to grow exponentially throughout the 40-day observation period, reaching a final RTV of approximately 10.58 (p < 0.001), indicating persistent tumor progression in the absence of treatment. MBZ monotherapy (10 mg/kg) delayed tumor expansion during the initial phase of treatment; however, tumor growth continued progressively throughout the study, reaching a final RTV of approximately 9.47. Although tumor progression remained slower than that observed in untreated controls, the difference between the two groups gradually narrowed during the later stages of the experiment. Gefitinib monotherapy produced greater and more sustained tumor growth inhibition than MBZ alone (p < 0.05). Tumor expansion remained consistently lower throughout the study, reaching a final RTV of approximately 6.71, indicating prolonged suppression of tumor progression. The combination of MBZ (10 mg/kg) and gefitinib (50 mg/kg) demonstrated the greatest and most durable antitumor activity. Tumor growth remained markedly suppressed throughout the 40-day treatment period, reaching a final RTV of approximately 4.55 (p < 0.001), representing the lowest tumor volume among all experimental groups. The separation between the combination group and the monotherapy groups became progressively more pronounced during the later stages of treatment, indicating sustained therapeutic efficacy during prolonged drug administration. Importantly, no obvious rebound acceleration of tumor growth was observed in the combination-treated animals during the study period. Overall, the long-term study demonstrated that combining low-dose MBZ with gefitinib maintained durable inhibition of tumor progression throughout extended treatment and provided superior tumor growth control compared with monotherapy (Fig 8).

**Fig 8:**
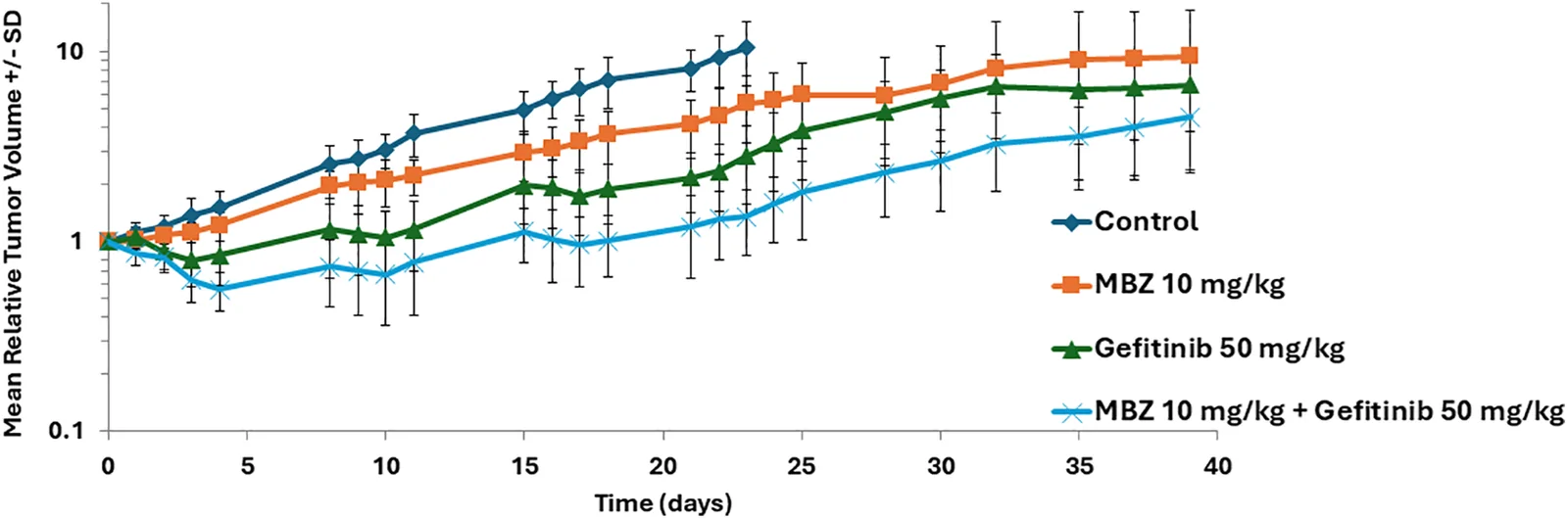
Long-term tumor growth kinetics following repeated dosing schedule.

Tumor growth kinetics were further evaluated by determining the median RTV2 and growth delay. Untreated tumors exhibited rapid growth, with a median RTV2 of 6.2 days. MBZ monotherapy prolonged the median RTV2 to 10.3 days, corresponding to a growth delay of 4.1 days compared with untreated controls (p < 0.05). Gefitinib further delayed tumor doubling to 15.5 days, resulting in a growth delay of 9.3 days (p < 0.01 versus control). The combination treatment produced the greatest suppression of tumor growth kinetics, extending the median RTV2 to 25 days and increasing tumor growth delay to 18.8 days (p < 0.01 versus control). These findings indicate that combined treatment markedly slowed tumor progression compared with either untreated or single-agent therapy.

Treatment tolerability was assessed by monitoring relative body weight throughout both short-term (11 days) and long-term (40 days) studies. Control animals exhibited gradual physiological weight gain during the experimental period. Body weight remained generally stable in all treatment groups, with only minor transient fluctuations during the early treatment phase. Gefitinib-treated mice showed a modest temporary reduction in body weight, whereas combination-treated animals exhibited a transient decrease of approximately 2–3%, which recovered during continued treatment. No sustained body weight loss or evidence of treatment-related systemic toxicity was observed in any group, indicating that prolonged administration of MBZ, gefitinib, and their combination was well tolerated (Figs 9 and 10; Table 1).

**Fig 9:**
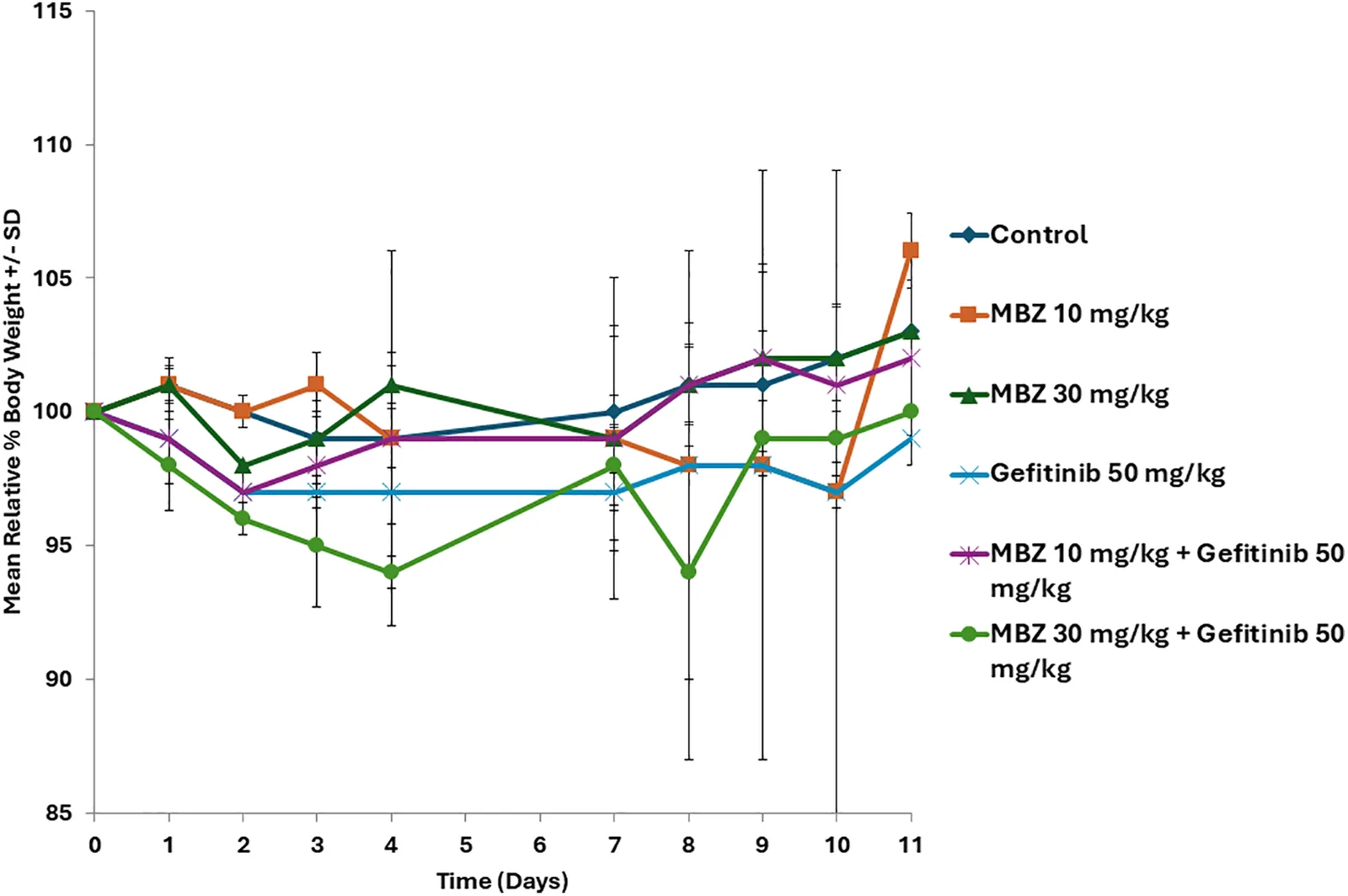
Body weight changes during short-term treatment in GR5 xenograft model.

**Fig 10:**
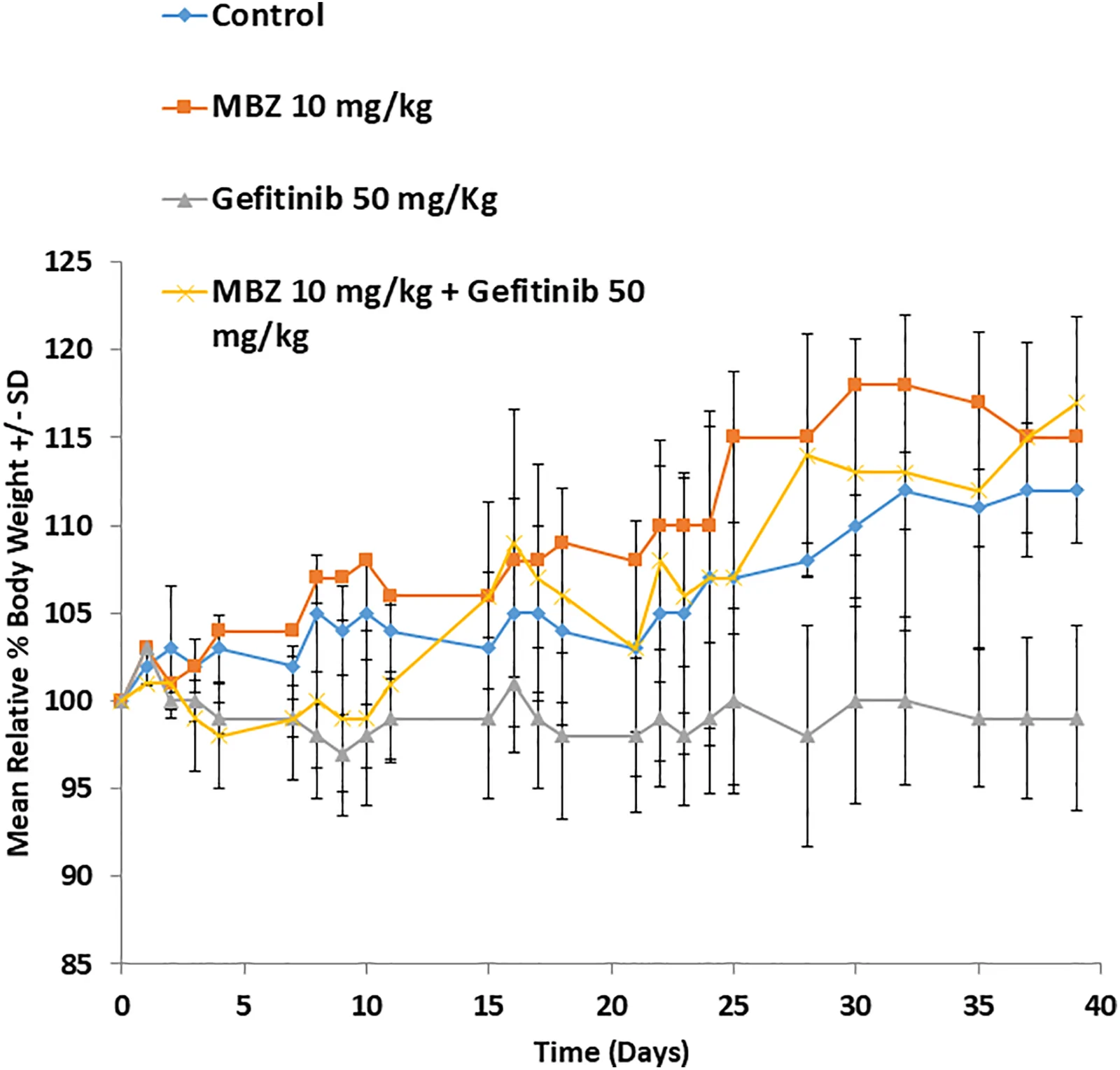
Long-term body weight profile during repeated treatment cycles.

**Table 1:** Tumor growth delay and tolerability analysis in GR5 xenograft-bearing mice.

Group	Median time to RTV2 (Days)	Growth delay (Days)	Significance	Maximum % weight loss
Untreated controls	6.2	–	–	0
MBZ 10 mg/kg; d0-4, 7–11, 15–18	10.3	4.1	p < 0.05	0
Gefitinib 50 mg/kg; d0-4, 7–11, 15–18	15.5	9.3	p < 0.01	3 (d9)
MBZ 10 mg/kg + Gefitinib 50 mg/kg; d0-4, 7–11, 15–18	25	18.8	p < 0.01	2 (d4)

Collectively, the *in vivo* studies demonstrate that both MBZ and gefitinib inhibited tumor progression in HCC827 GR5 xenografts, with the magnitude of inhibition varying according to treatment regimen. MBZ alone produced dose-dependent antitumor activity during the short-term study, whereas gefitinib provided greater tumor growth inhibition than the lower MBZ dose during prolonged treatment. The combination regimen consistently achieved the greatest suppression of tumor growth in both short-term and long-term experiments, maintaining tumor volumes substantially below those observed in all monotherapy groups. Furthermore, combination treatment produced the longest tumor doubling time while maintaining excellent tolerability, supporting the enhanced therapeutic efficacy of low-dose MBZ in combination with gefitinib in this gefitinib-resistant xenograft model.

## 4. Discussion

The present study provides an integrated mechanistic framework in which dual targeting of EGFR signaling and microtubule dynamics is associated with enhanced replication-associated cellular stress and sustained growth suppression in gefitinib-resistant NSCLC which we interpret as consistent with impaired replication-stress tolerance. Using the clinically relevant HCC827 GR5 model, we demonstrate that therapeutic resistance is not merely a consequence of persistent EGFR pathway activation or secondary mutation-driven escape but rather reflects a stabilized adaptive state in which tumor cells actively maintain replication fork integrity under continuous oncogenic and pharmacological stress pressure. Within this framework, survival is determined not by single pathway dependency but by the capacity to preserve replication system coherence under dual-axis perturbation.

This study makes several novel conceptual advancements, including our first repositioning of EGFR-TKI resistance as a replication-stress buffered survival phenotype rather than a purely signaling-driven escape mechanism. Minimal responses to gefitinib were observed in all endpoints measured here for the gefitinib resistant HCC827 GR5 cells, which as predicted above, are unable to be stalled in cell cycle by inhibition of the signaling pathway that drives their proliferation. Instead, their proliferation is clearly driven by a replication-stress buffered survival phenotype that is partially decoupled from their EGFR signaling pathway, which can be partially and/or conditionally inhibited by EGFR-TKIs such as gefitinib. This decoupling and consequent buffering of the effects of such EGFR inhibition on replication stress is likely mediated by a set of adaptive and/or compensatory changes that allow the tumor cells to continue to synthesize DNA and thus to enter and complete cell division. These changes could include increased checkpoint tolerance, activation of new signaling bypasses around the EGFR signaling pathway, and/or increased stability of the DNA replication machinery, thereby providing a replication-stress buffered survival phenotype that is able to maintain proliferation in the face of continued EGFR inhibition.

In contrast, the anti-parasitic compound MBZ induced a significantly different, more orthogonal type of stress. It targets β-tubulin of microtubules to cause architectural disruptions of these structures. However, in cancer cells, microtubules not only are required for mitosis, but they are also involved in the intracellular transport of a large variety of replication and cell cycle associated proteins [41,42]. Thus, microtubules are involved in organizing and stabilizing sites of active replication, or replication factories. In addition, the cell cycle progression from G1 to S and through S phase is coordinated and supported by the microtubule structures of the cell [43–45]. Thus, as MBZ caused dose-dependent decrease in the viability of the cancer cells, the threshold-based structural collapse of these diverse structures involved in replication and cell cycle also caused a structural type of replication stress that could affect the integrity of replication in interphase cells.

Of note, also partial interference with microtubule dynamics is sufficient to cause sensitization of gefitinib-resistant cancer cells for a combined treatment with gefitinib and MBZ. Doses of MBZ that are several-fold below the dose that on its own are causing a considerable reduction in cell viability induce a cytotoxic effect in combination with gefitinib that is like that of much higher doses of MBZ. This finding points to a critical threshold of damage of replication structures and a subsequent collapse of the replication machinery that can be caused by the interference with microtubule dynamics in gefitinib-resistant cancer cells. Importantly, these findings further support a functional link between the EGFR signaling pathway and the microtubule system and indicate that a targeted disruption of both axes of resistance causes a failure of replication stress buffering that cannot be overcome by resistant cancer cells [46–48].

The comet assay provided insight into the replication stress-DNA damage relationship. Early in the treatment course SSBs predominated, representing replication fork stalling. Gefitinib treatment induced modest increases in SSBs, consistent with its failure to perturb replication fork stability in this resistant cancer cell model. Replication stress activated checkpoints presumably are reinforced to protect the replication machinery from perturbation by the constitutively active EGFR signaling pathway [49–51]. Notably, treatment with MBZ resulted in a marked increase in SSBs in a dose-dependent manner. This indicates that MBZ causes a replication-associated stress response that is independent of typical DNA-damage pathways. In contrast to gefitinib, at high concentrations of MBZ the signal for SSBs was even decreased. However, this decrease in SSBs is not a sign of relief from genomic stress, rather it is a result of the fact that SSBs are no longer a stable and detectable intermediate of replication fork stalling. Instead, these structures are progressively collapsed into more stable and less detectable forms of DNA damage.

Neutral comet assay showed that double-strand breaks, indicated by Comet Tail Moment Neutral, increased with high dose of MBZ, suggesting that under prolonged treatment cells encountered replication fork collapse and irreversible genomic damage. Notably, while the combination group showed an intermediate level of double-strand breaks, its extreme cytotoxicity suggested that the threshold for lethal cell damage was achieved prior to the highest level of DNA fragmentation. Notably, while γ-H2AX is typically used as a surrogate for DSBs, this marker reflects the level of chromatin-associated signaling, and therefore, can report the level of total DNA-stress, including replication stress [52–56]. Thus, while MBZ-induced DNA-stress elicited a dose-dependent increase in γ-H2AX, indicating the activation of the ATM/ATR pathway in response to formation of stalled or broken replication forks, combination therapy elicited relatively low levels of γ-H2AX-signaling, consistent with a model in which the replication stress response is collapsed and cell cycle arrested in a state of checkpoint exhaustion, leading to lethality of the treated cells.

In summary, our *in vitro* study revealed that in gefitinib-resistant NSCLC cells replication stress homeostasis is collapsed by the combined effects of EGFR signaling and microtubule dynamics. Consequently, here replication stress homeostasis failure is identified as a critical and new vulnerability axis in cancer therapy. Therefore, our study demonstrates that the cytotoxicity of anticancer drugs does not correlate with the amount of DNA damage but rather with the degree of replication stress-induced cellular collapse. Thus, γ-H2AX signaling can be considered as a stress-response amplifier rather than a quantitative marker of cell death.

The *in vivo* studies on HCC827 GR5 xenografts supported our findings from cell line studies. Combination of gefitinib with low-dose MBZ led to a more marked decrease in tumor growth than either agent given alone or induced a more sustained effect than high-dose MBZ alone. In terms of short-term pharmacodynamic effects, gefitinib or MBZ, or their combination, inhibited tumor growth by inducing a replicative stress-adaptive stabilized tumor growth state or by killing the tumor cells. Importantly, the long-term effect of the combination treatment resulted in a replication-limited equilibrium or a subcritical growth state of the remaining tumor cells that lacked exponential growth and continued to grow at a slow rate, indicating that surviving tumor cells had been repopulated by a small number of cells of reduced fitness.

Gefitinib caused a temporary decrease in tumor growth of approximately 50% after 12 days which then reached a plateau phase and levelled off. This finding supports the notion that resistant EGFR-addicted tumors can recover from EGFR-inhibition by re-establishing their proliferative programs. In contrast, MBZ caused a complete and sustained suppression of tumor growth which reached a plateau phase of approximately 100 mm³ after 12 days. Thus, MBZ treated tumors are not only growth suppressed but also restricted from re-growth in contrast to gefitinib-treated tumors. The combination arm induced long term growth suppression that differed from the single agents, producing a plateaued phase of growth suppression without subsequent increase in tumor size. This non-growing state was quantified by a statistically significant decrease in tumor doubling time indicating that growth suppressed by combination therapy is of a quality that does not allow tumor to re-grow (sub-critical growth).

As for the doubling time of the tumors, the present study demonstrated a significant prolongation of this parameter in the tumors treated with the combination of gefitinib and MBZ, compared to both monotherapies. More importantly, this increase in doubling time indicated that the ability of the tumor cells to complete their cell cycle had been dramatically impaired. Therefore, while the combination of gefitinib and MBZ led to a marked decrease in the number of tumor cells, it also caused a profound decrease in the tumor cells’ ability to proliferate. This indicated that the tumor cells that survived the treatment were not only fewer in number, but also functionally impaired, in terms of their ability to complete their cell cycle under continued stress.

An important insight from these results is that temporary collapse of replication in resistant NSCLC can be translated into long-term growth inhibition by allowing sufficient time for a tumor to exhaust its repertoire of mechanisms that allow it to survive under persistent oncogenic and therapeutic stress. In summary, combining EGFR inhibition with a drug that temporarily disrupts DNA replication, such as MBZ, can produce sustained growth inhibition in resistant tumors. This effect appears to result from the transient induction of replication stress, which is followed by long-term reprogramming of tumor cell growth, even in cells that had previously stabilized in a resistant state. The integrated data presented within this study supports a three-axis system of vulnerability for cancer cells [57–60]. These three axes of vulnerability include the signaling for cell growth (the EGFR pathway), the structural basis for the replication of the cells (the microtubules) and the replication stress response system (the network of checkpoint controls that maintain genomic integrity during periods of elevated oncogenic stress).

A systems-level perspective further underscores the potential advantage of combining two drugs that individually target different aspects of tumor-cell biology, here EGFR signaling and microtubule dynamics. Gefitinib inhibits EGFR-dependent signaling, whereas MBZ perturbs microtubule-dependent cellular processes, potentially creating complementary cellular stresses in gefitinib-resistant cancer cells. Under the conditions tested, the combination produced a greater reduction in cellular viability and enhanced DNA-damage-associated responses than either agent alone, suggesting that simultaneous perturbation of these biological processes can increase the vulnerability of gefitinib-resistant cells. However, because the present study did not employ a formal pharmacological synergy model, such as combination-index, Bliss independence, Loewe additivity, or response-surface analysis, the observed interaction is more appropriately described as an enhanced combination effect rather than formally quantified synergism. The integrated molecular, cellular, and *in vivo* findings therefore support a model in which concurrent EGFR inhibition and microtubule disruption increases cellular stress and reduces the capacity of resistant tumor cells to maintain proliferative activity, while the precise molecular sequence linking these perturbations to sustained tumor-growth suppression requires further investigation.

These results contrast with our previous study using EGFR-independent models (A549 and MDA-MB-231) where we reported the induction of generalized cytotoxicity and inflammation following combination of EGFR and microtubule inhibitors [26]. The present study suggests that, in EGFR-dependent resistant models, complementary biological effects of EGFR inhibition and microtubule disruption may contribute to the enhanced antitumor effect observed with combination treatment. This study, by employing molecular, cellular and *in vivo* based tumor growth kinetics endpoints, provides a multi-scale, integrative framework of the EGFR-addicted NSCLC resistant to EGFR-TKIs and reveals that replication stress homeostasis failure, formed by a three-axis system of EGFR signaling, microtubule architecture, and replication stress response structures, is a critical and previously underappreciated vulnerability axis that can be targeted by combination therapy with gefitinib and MBZ to kill the tumor cells. In addition, this study offers insights into the complex processes of short- and long-term tumor growth suppression following the withdrawal of therapy, which is of importance for future clinical trials with a focus on tumor growth kinetics. The present study has certain limitations. Lack of data on direct tracking of replication forks (e.g., by DNA fiber assays or by single-molecule imaging of replicating chromosomes) hampers quantification of frequency of stalled forks, and their subsequent restart or collapse. Importantly, the present study did not directly quantify replication-fork progression, fork protection, fork restart, or checkpoint exhaustion. Accordingly, the proposed replication-stress model should be regarded as a mechanistic interpretation supported by convergent viability, comet-assay, γ-H2AX, and tumor-growth data rather than as direct demonstration of a specific replication-fork lesion. The HCC827 GR5 cell line has been previously shown to harbor gefitinib-resistant phenotypes of clinical origin. Yet, in the experiments presented above, all aspects of gefitinib-resistant phenotype of HCC827 GR5 cells were not addressed. Some other resistance mechanisms not covered in the present study, such as those mediated by activation of MET pathway, by histological reprogramming of NSCLC, or by adaptation of tumor cells to anti-cancer therapy in the tumor stroma, need to be tested for their response to the dual therapy with EGFR and microtubule-targeting drugs. These additional experiments should be performed using a panel of patient-derived xenografts (PDXs) and models of multi-clonal resistant NSCLC. Despite these limitations, this study generates sufficient data to redefine EGFR-TKI resistance to replication stress homeostasis failure and establish a rational approach to develop combination therapies that target several axes of a tumor network to collapse its adaptability in resistant NSCLC. Ultimately, the integration of findings from the present study provides a sound framework to predict the requirement for sustained replication stress-induced dysfunction in resistant NSCLC by targeting the interdependent signaling for cell growth, structural support of replication and stability of replication stress response, here referred to as the three-axis vulnerability model. This novel model therefore outlines future perspectives for combination therapy in the management of EGFR-addicted NSCLC after failure of monotherapy.

## 5. Conclusion

The present study demonstrates that combining gefitinib with MBZ enhances loss of viability and suppresses tumor growth in the gefitinib-resistant HCC827 GR5 model. Gefitinib alone produced limited activity, whereas MBZ induced concentration-dependent cytotoxicity and DNA-damage-associated responses. Importantly, low-dose MBZ combined with gefitinib produced a substantially greater reduction in cellular viability and a more durable inhibition of xenograft growth than either monotherapy under the conditions tested. Comet-assay and γ-H2AX findings support increased replication-associated cellular stress and DNA-damage-response signaling, although direct replication-fork assays and checkpoint measurements will be required to define the precise molecular sequence underlying this response. The findings therefore provide preclinical evidence that simultaneous perturbation of EGFR signaling, and microtubule dynamics may increase the vulnerability of gefitinib-resistant EGFR-mutant NSCLC cells and support further investigation of MBZ as a potential adjunct to EGFR-targeted therapy.

## Supporting information

S1 File Anti-γH2Ax blot (17 kDa, rabbit, 1:3000 dilution in 5% BSA). (PDF)
