# Supplementary material for: Dual targeting of EGFR signaling and microtubule dynamics enhances replication-associated cellular stress and suppresses tumor growth in gefitinib-resistant non-small cell lung cancer
Source: PLoS One. 2026 Oct 8;21(10):e0360164. doi: 10.1371/journal.pone.0360164 (PMC13648989; doi:10.1371/journal.pone.0360164)
Supplement: Supplementary file 1 [file pone.0360164.s001.pdf]

Anti-γH2Ax blot (17 kDa, rabbit, 1:3000 dilution in 5% BSA)

1 2 3 4 5

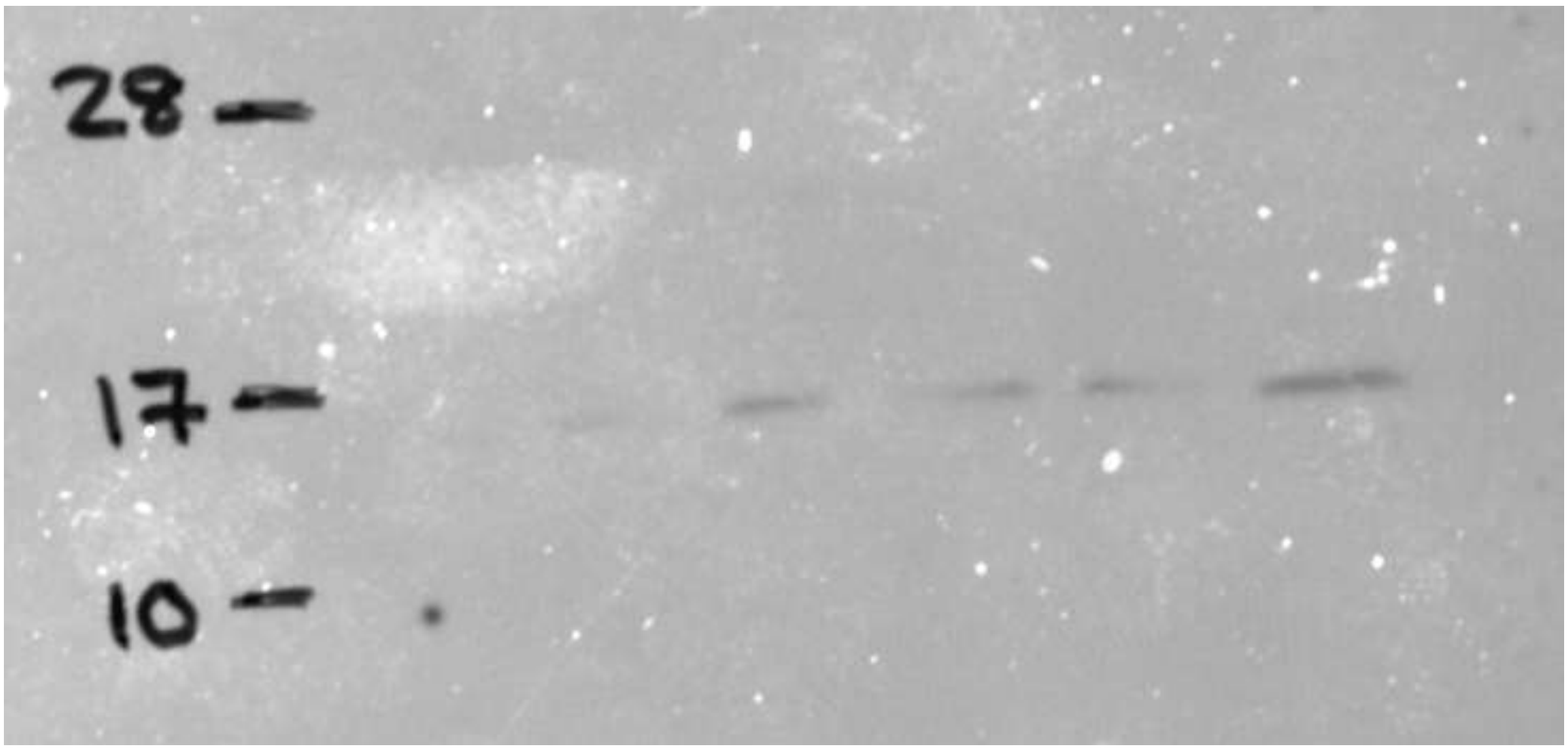

Lane 1: Control

Lane 2: Gefitinib 1 µM

Lane 3: Mebendazole 0.1 µM

Lane 4: Mebendazole 0.5 µM

Lane 5: Gefitinib 1 µM + Mebendazole 0.1 µM
